# Causal role of immune cells in allergic rhinitis: A bidirectional Mendelian randomization study

**DOI:** 10.1016/j.bjorl.2025.101726

**Published:** 2025-10-24

**Authors:** Yuhan Tan, Xiaoyan Hu, Jing Zhang, Shuoyu Wan, Liang Jiang

**Affiliations:** a Affiliated Hospital of Southwest Medical University, Department of Otolaryngology Head and Neck Surgery, Luzhou City, People's Republic of China; b Southwest Medical University, School of Basic Medicine, Department of Pathogen Biology, Luzhou City, People's Republic of China; c Southwest Medical University, Public Center of Experimental Technology of Pathogen Biology Technology Platform, Luzhou City, People's Republic of China

**Keywords:** Allergic rhinitis, Immune cells, Mendelian randomization, Causal role

## Abstract

**Objective:**

Previous observational studies have indicated an association between various immune cells and allergic rhinitis; however, the causal relationship remains uncertain. This study seeks to further investigate the causal link between immune cells and allergic rhinitis through bidirectional two-sample Mendelian randomization analysis.

**Methods:**

Allergic rhinitis related data were obtained from the MRC-IEU database, while data for 731 immune cells were sourced from the GWAS catalog. The causal association between immune cells and allergic rhinitis was assessed using inverse variance weighting, MR-Egger, weighted median, simple mode, and weighted mode methods. Stability and reliability of the findings were evaluated through Cochran's *Q* test, MR-Egger intercept p-value, and leave-one-out analysis.

**Results:**

Primarily based on IVW methods, 38 distinct immunocyte phenotypes were identified as potentially influencing allergic rhinitis (p < 0.05), while allergic rhinitis was found to potentially impact 20 different immunocyte phenotypes (p < 0.05). The results for heterogeneity, horizontal pleiotropy, and sensitivity analyses demonstrated stability.

**Conclusion:**

This study establishes a strong genetic correlation between immune cells and allergic rhinitis, enhances current knowledge regarding the role of immune cells in AR, and offers potential targets for the development of novel immune-based therapeutic strategies.

**Level of evidence:**

Level 5.

## Highlights

• Immune cells causally affect allergic rhinitis, guiding therapy and clinical study.
• The study used two independent samples to avoid overfitting caused by duplication.
• Bidirectional Mendelian randomization analysis effectively excluded reverse causality.
• Multiple immune cells were evaluated for AR association, preventing single-marker bias.

## Introduction

Allergic Rhinitis (AR) is a non-infectious inflammatory condition mediated by Immunoglobulin E (IgE). Exposure to allergens such as dust mites, grass pollen, and animal dander in atopic individuals can trigger symptoms including nasal congestion, paroxysmal sneezing, and watery rhinorrhea.^1^ According to a study, AR affects about 5%–50% of the global population, with its prevalence continuing to rise, significantly impairing sleep quality and overall well-being.^2^ Current treatment approaches, including allergen avoidance, pharmacotherapy, and immunotherapy, often do not achieve the expected curative outcomes. The conventional perspective suggests that an imbalance between innate and adaptive immunity plays a key role in the pathogenesis of AR.^3^ Therefore, investigating the causal association between immune cells and AR is essential for facilitating early diagnosis and advancing novel therapeutic strategies.

A series of immune responses triggered by allergen exposure in atopic individuals play a vital role in the occurrence and progression of AR. Dendritic Cells (DCs), T-cells, basophils, and mast cells are key immune cell types involved in the onset and progression of AR. During initial allergen exposure, IgE binds to the FcεRI receptor on the surface of basophils and mast cells. Upon subsequent exposure, allergens interact with the Fab region of IgE molecules on these cells, inducing the release of histamine, leukotrienes, and other inflammatory mediators, leading to nasal mucosa inflammation.^4^ Different T-cell subtypes contribute significantly to immune response and inflammation in AR. Helper-T (Th) cells are classified into Th1 and Th2 subtypes. While Th1 secretes cytokines to enhance cellular immunity, Th2 promotes B-cell activation for humoral immunity by secreting Th2 cytokines and inhibiting Th1 proliferation. An imbalance favoring Th2 over Th1 can intensify allergic reactions.^5^^,^^6^ Th17 cells, derived from CD4+ T-cells, can secrete cytokines involved in inflammatory responses to further exacerbate AR.^7^ Research has shown that regulatory T-cells (Tregs) exert inhibitory effects on B-cell activity and CD4+ T-cell function, thereby suppressing immune responses.^8^ A decrease in Tregs numbers weakens this suppression, leading to sustained T-cell activation, which disrupts immune homeostasis in AR.^9^^,^^10^ A recent study has demonstrated a close association between the imbalance of Tregs and Th17 cells and the onset of AR.^11^ However, the relationship between AR and immune responses remains complex, so that further research is required to elucidate the causal relationship between immune cells and AR.

Mendelian Randomization (MR) is a statistical approach based on Mendelian inheritance laws.^12^ This method utilizes genetic variants that exhibit strong correlations with exposure levels as Instrumental Variables (IVs) to investigate the causal relationship between exposure and clinically relevant outcomes.^13^^,^^14^ In this study, a two-sample bidirectional MR analysis was conducted to integrate large-scale genetic data with immune cell traits, aiming to assess the causal relationship between 731 immune cell traits and AR.

## Methods

### Study design

**Fig. 1:**
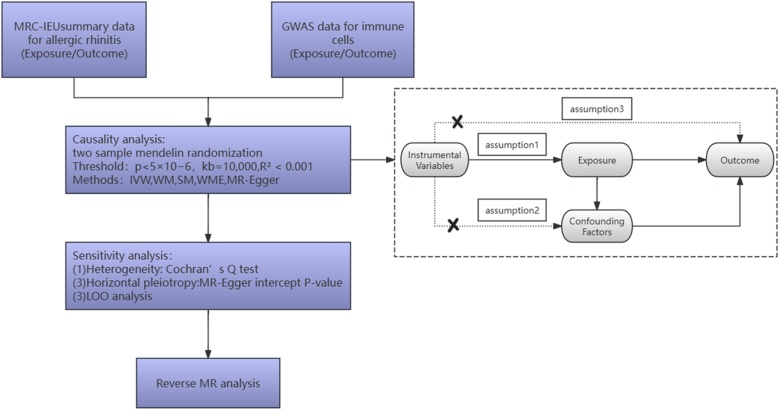
Study design and three assumptions of MR. GWAS, Genome-Wide Association Study; IVW, Inverse Variance Weighting; WM, Weighted Median; SM, Simple Mode; WME, Weighted Mode; LOO, Leave-One-Out; MR, Mendelian Randomization.

A two-sample bidirectional MR analysis was conducted to examine the causal relationship between 731 immune cell traits and AR. Single Nucleotide Polymorphisms (SNPs) associated with these immune cell traits were utilized as IVs. To ensure the validity and reliability of the results, effective IVs as risk factors were required to satisfy three assumptions in MR analysis. The detailed flow chart of the study design and the above assumptions are presented in Fig. 1.

### Data sources for allergic rhinitis

Summary data for AR were obtained from the MRC-IEU database. This dataset included a sequencing depth of 9,587,836 SNPs and comprised individuals of European descent, with 27,415 cases and 457,183 controls (https://gwas.mrcieu.ac.uk/datasets/ebi-a-GCST90038664/). All participants shared a common genetic background as they were of European ancestry.

### Data sources for immune traits

The summary statistics of SNPs associated with 731 immune traits were extracted from the Summary-level Genome-Wide association Study (GWAS) catalog (accession numbers from GCST0001391 to GCST0002121),^15^ which is publicly available. By analyzing peripheral blood samples from 3,757 Sardinian individuals, the study identified about 22 million genetic variations across 731 immune cell traits. The total immunophenotypes included absolute cel counts (n = 118), median fluorescence intensities representing surface antigen levels (n = 389), morphological parameters (n = 32), and relative cell counts (n = 192).

### Selection of instrumental variables

Optimal IVs were selected based on rigorous screening criteria to ensure the reliability and accuracy of the findings. Briefly, summary-level GWAS statistics on immune cells were screened, and SNPs with a significance threshold of p < 5 × 10^−6^ were selected.^16^ Following the independence assumption of MR, a coefficient R2 of 0.001 and clump distance of 10,000 kb maximized their efforts to avoid any potential linkage disequilibrium IVs.^17^ Additionally, palindromic SNPs were excluded to ensure consistent allele alignment between the immune cell group and the AR group. The process of selecting optimal IVs is illustrated in Fig. 1.

### Statistical analysis

Multiple analytical methods were utilized to determine whether immune traits have a causal relationship with the risk of AR, including Inverse Variance Weighting (IVW), MR-Egger, weighted median, simple mode, and weighted mode. Among these, IVW served as the primary method. Cochran’s *Q* test was conducted to evaluate heterogeneity in the IVW model, with a p-value <0.05 indicating the presence of heterogeneity. The MR-Egger regression intercept was used to assess pleiotropy, where a nonzero intercept with a p-value <0.05 suggested the existence of horizontal pleiotropy. Additionally, a leave-one-out analysis was performed to determine whether the estimates were influenced by a single SNP, ensuring the robustness of the findings.

## Results

### The effect of immune cell phenotypes on allergic rhinitis

**Fig. 2:**
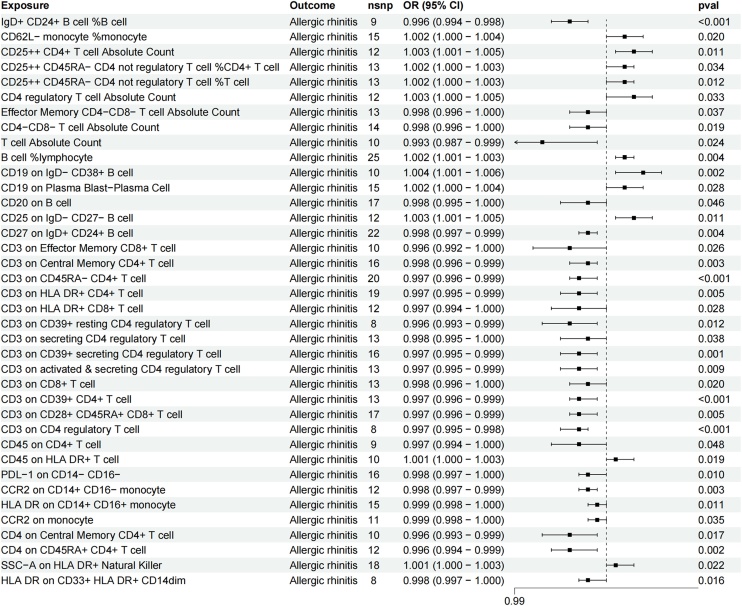
The forest plot shows the causal relationship between immune cell phenotypes and allergic rhinitis.

At a significance threshold of 0.05, 38 immune cell phenotypes were identified as potentially associated with AR (Fig. 2).

Regarding absolute cell counts, an increase in Effector Memory CD4-CD8- T-cell Absolute Count (OR = 0.998 [0.996, 0.999], p = 0.037), CD4-CD8- T-cell Absolute Count (OR = 0.998 [0.996, 0.999], p = 0.019), and T-cell Absolute Count (OR = 0.993 [0.987, 0.999], p = 0.024) was associated with a decreased risk of AR. Conversely, an increase in CD25++ CD4+ T-cell Absolute Count (OR = 1.003 [1.001, 1.005], p = 0.011) and CD4 regulatory T-cell Absolute Count (OR = 1.003 [1.0002, 1.005], p = 0.033) was linked to a higher risk of AR.

For relative cell counts, an increase in IgD+ CD24+ B-cell %B-cell (OR = 0.996 [0.994, 0.998], p = 0.0005) was associated with a reduced risk of AR. However, a higher proportion of CD62L− monocyte %monocyte (OR = 1.002 [1.0003, 1.0037], p = 0.020), CD25++ CD45RA− CD4 not regulatory T-cell %CD4+ T-cell (OR = 1.002 [1.0001, 1.003], p = 0.034), CD25++ CD45RA- CD4 not regulatory T-cell %T cell (OR = 1.002 [1.0004, 1.003], p = 0.012), and B-cell %lymphocyte (OR = 1.002 [1.001, 1.003], p = 0.0039) was associated with an increased risk of AR.

Regarding median fluorescence intensity, increased levels of CD19 on IgD- CD38+ B-cell (OR = 1.004 [1.001, 1.006], p = 0.002), CD19 on Plasma Blast-Plasma Cell (OR = 1.002 [1.000, 1.004], p = 0.028), and CD25 on IgD- CD27- B-cell (OR = 1.003 [1.001, 1.005], p = 0.011) correlated with a higher incidence of AR. In contrast, elevated levels of CD20 on B-cell (OR = 0.998 [0.995, 1.000], p = 0.046), CD27 on IgD+ CD24+ B-cell (OR = 0.998 [0.997, 0.999], p = 0.004), CD3 on Effector Memory CD8+ T-cell (OR = 0.996 [0.993, 0.999], p = 0.026), CD3 on Central Memory CD4+ T-cell (OR = 0.998 [0.996, 0.999], p = 0.003), CD3 on HLA DR+ CD4+ T-cell (OR = 0.997 [0.995, 0.999], p = 0.005), CD3 on HLA DR+ CD8+ T-cell (OR = 0.997 [0.994, 0.999], p = 0.028), CD3 on CD39+ resting CD4 regulatory T-cell (OR = 0.996 [0.993, 0.999], p = 0.012), CD3 on secreting CD4 regulatory T-cell (OR = 0.998 [0.996, 1.000], p = 0.038), CD3 on CD39+ secreting CD4 regulatory T-cell (OR = 0.997 [0.995, 0.999], p = 0.001), CD3 on activated & secreting CD4 regulatory T-cell (OR = 0.997 [0.995, 0.999], p = 0.009), CD3 on CD8+ T-cell (OR = 0.997 [0.995, 0.999], p = 0.020), CD3 on CD39+ CD4+ T-cell (OR = 0.997 [0.995, 0.999], p = 0.0002), CD3 on CD28+ CD45RA+ CD8+ T-cell (OR = 0.997 [0.995, 0.999], p = 0.005), CD3 on CD4 regulatory T-cell (OR = 0.997 [0.995, 0.999], p = 0.0001), CD45 on CD4+ T-cell (OR = 0.998 [0.996, 1.000], p = 0.048), CD4 on Central Memory CD4+ T-cell (OR = 0.998 [0.996, 1.000], p = 0.048), CD4 on CD45RA+ CD4+ T-cell (OR = 0.996 [0.994, 0.999], p = 0.040), PDL-1 on CD14- CD16- (OR = 0.998 [0.997, 1.000], p = 0.011), CCR2 on CD14+ CD16- monocyte (OR = 0.998 [0.997, 0.999], p = 0.003), HLA DR on CD14+ CD16+ monocyte (OR = 0.999 [0.998, 1.000], p = 0.011), CCR2 on monocyte (OR = 0.999 [0.998, 1.000], p = 0.035), and HLA DR on CD33+ HLA DR+ CD14dim (OR = 0.998 [0.997, 1.000], p = 0.016) were associated with a reduced risk of AR.

Regarding morphological parameters, an increase in SSC-A on HLA DR+ Natural Killer (OR = 1.001 [1.000, 1.003], p = 0.022) correlated with an elevated risk of AR.

The findings from the remaining methods and sensitivity analyses further supported the robustness of the observed causal associations. The MR-Egger intercept analysis did not indicate the presence of horizontal pleiotropy.

### The effect of allergic rhinitis on immune cell phenotypes

**Fig. 3:**
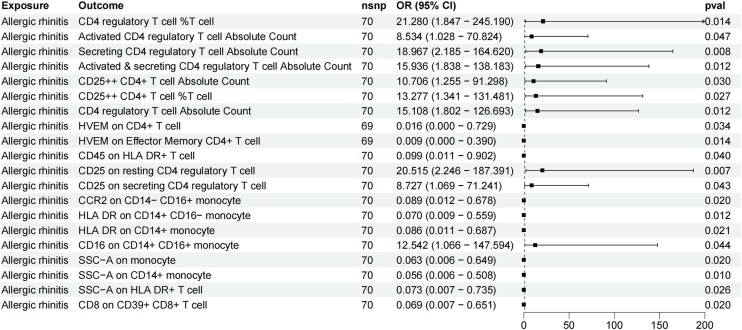
The forest plot shows the causal relationship between allergic rhinitis and immune cell phenotypes.

In the reverse MR analysis, at a significance level of 0.05, 20 immune cell phenotypes were identified as being influenced by AR (Fig. 3).

Regarding absolute cell counts, AR was associated with an increase in Activated CD4 regulatory T-cell Absolute Count (β = 2.14 [0.03, 4.26], p = 0.047), Secreting CD4 regulatory T-cell Absolute Count (β = 2.94 [0.78, 5.10], p = 0.008), Activated & Secreting CD4 regulatory T-cell Absolute Count (β = 2.77 [0.61, 4.93], p = 0.012), CD25++ CD4+ T-cell Absolute Count (β = 2.37 [0.23, 4.51], p = 0.030), and CD4 regulatory T-cell Absolute Count (β = 2.71 [0.59, 4.84], p = 0.012).

Regarding absolute cell counts, AR was associated with an increase in CD25++ CD4+ T-cell %T-cell (β = 2.58 [0.29, 4.88], p = 0.027) and CD4 regulatory T-cell %T-cell (β = 3.06 [0.61, 5.50], p = 0.014).

Regarding median fluorescence intensity, AR was linked to a decrease in HVEM on CD4+ T-cell (β = −4.11 [−7.91, −0.32], p = 0.034), HVEM on Effector Memory CD4+ T-cell (β = −4.66 [−8.37, −0.94], p = 0.014), CD45 on HLA DR+ T-cell (β = −2.32 [−4.53, −0.10], p = 0.040), CCR2 on CD14− CD16+ monocyte (β = −2.42 [−4.45, −0.39], p = 0.020), HLA DR on CD14+ CD16− monocyte (β = −2.66 [−4.73, −0.58], p = 0.012), HLA DR on CD14+ monocyte (β = −2.45 [−4.53, −0.38], p = 0.021), and CD8 on CD39+ CD8+ T-cell (β = −2.67 [−4.92, −0.43], p = 0.020). Conversely, AR was associated with an increase in CD25 on resting CD4 regulatory T-cell (β = 3.02 [0.81, 5.23], p = 0.007), CD25 on Secreting CD4 regulatory T-cell (β = 2.17 [0.07, 4.27], p = 0.043), and CD16 on CD14+ CD16+ monocyte (β = 2.53 [0.06, 4.99], p = 0.044).

Regarding morphological parameters, AR resulted in a decrease in SSC-A on monocyte (β = −2.77 [−5.11, −0.43], p = 0.020), SSC-A on CD14+ monocyte (β = −2.87 [−5.07, −0.68], p = 0.010), and SSC-A on HLA DR+ T-cell (β = −2.62 [−4.93, −0.31], p = 0.026).

The results obtained from additional methods and sensitivity analyses further supported the robustness of the observed causal relationships.

## Discussion

We explored potential causal relationships between 731 immune cell phenotypes and AR using publicly available genetic data. In the analysis assessing the impact of immune cell phenotypes on AR, 11 immune cell phenotypes were positively associated with the risk of AR, and 27 immune cell phenotypes were negatively associated with it.

While CD8+ T-cells have been primarily associated with anti-tumor immunity, recent studies suggest their involvement in immune responses related to AR.^18^ For example, Zhengjie Chen et al. reported that an increased ratio of Naive CD8+ T-cells to the total CD8+ T-cell population may reduce susceptibility to AR.^19^ Additionally, CD8+ Tregs are recognized as a heterogeneous subset of CD8+ T-cells capable of regulating immune responses by targeting antigen-activated CD4+ T-cells.^20^ Immune-activated CD8+ Tregs are considered as essential participants in maintaining immune homeostasis. An experiment conducted by Lin Lin et al. demonstrated that the transfer of CD8+ Tregs into AR mouse models resulted in a reduced inflammatory response,^21^ supporting the hypothesis that CD8+ Tregs exert an inhibitory effect on AR progression. Based on these findings, it is suggested that CD8+ T-cells may have a potential immunosuppressive role in AR. As a type 1 transmembrane protein tyrosine phosphatase, CD45 expressed in hematopoietic and immune system cells. Among its subtypes, those containing only one exon are classified as RA or RB. Previous research has demonstrated that CD45 plays both inhibitory and activating roles in T-cell regulation through the dephosphorylation of activating and inhibitory phosphotyrosine residues. On one hand, the depletion of CD45 leads to the abolition of TCR-mediated signaling. On the other hand, CD45 exhibits substantial phosphatase activity, preventing non-specific or excessive activation of immune cells.^22^ CD3 is a polymeric protein complex shared by T-cells, and the CD3/TCR complex functions as a complete signaling unit essential for mediating signal transduction, T-cell proliferation, and inhibitory responses.^23^ The CD3/TCR complex can regulate T-cell activation intensity through an endosomal recycling mechanism.^24^ Consequently, the expression level of CD3 is indicative of T-cell activation intensity. Findings from this study indicate that increased CD3 expression on CD28+ CD45RA+ CD8+ T-cells is associated with a reduced risk of AR. Although limited research has explored the relationship between CD3 expression levels and CD8+ T-cell activation and function, the existing evidence provides novel insights. Based on these findings, it is speculated that increased CD3 expression may enhance the immunosuppressive function of a specific CD8+ T-cell subset via CD3/TCR signal transduction, thereby contributing to the inhibition of the immune response in AR.

PD-1 is a receptor expressed on T-cells,^25^ while its ligand, PDL-1, is primarily expressed on tumor cells and Antigen-Presenting Cells (APCs).^26^ The interaction between PD-1 and PDL-1 inhibits T-cell stimulation during immune responses, reducing T-cell activity, preventing excessive activation, proliferation, and autoimmune damage.^27^ Previous studies have shown that increased expression of PD-1 and PDL-1 not only directly suppresses CD4+ T-cell proliferation^28^ but also induces the conversion of naive CD4+ T-cells into Tregs by downregulating Akt, mTOR, and ERK2 while upregulating PTEN. This enhances the regulatory function of Tregs, further inhibiting the activation and proliferation of inflammatory cells.^29^ Specifically, the PD-1/PDL-1 pathway has been shown to suppress Th2 cell activation and reduce the secretion of pro-inflammatory cytokines such as IL-4, IL-5, and IL-13, thereby mitigating the inflammatory response in AR. Additionally, PD-1 is expressed by apoptotic cells, and its overexpression may enhance classical programmed cell death, leading to a reduction in inflammatory cell numbers through apoptosis, ultimately lowering the risk of AR.^30^ Findings from this study indicate that increased expression of PDL-1 on IL14-IL16- is negatively correlated with AR risk, supporting this regulatory mechanism.

HLA-DR is a key component of the human major histocompatibility complex Class II molecules, predominantly expressed on APCs.^31^^,^^32^ It plays a crucial role in antigen presentation and immune regulation. Studies have shown that when Natural Killer (NK) cells undergo functional activation or proliferation, the expression of several surface markers and receptors increases, including CD69, NKP4 and HLA-DR.^33^ The HLA-DR+ NK cell subset has been identified in immune-active organs such as the spleen, liver, blood, and lymph nodes of healthy individuals.^34^ Furthermore, several evidences suggest that the proportion of peripheral blood NK cells expressing HLA-DR increases in tuberculosis, HIV infection, and autoimmune diseases.^35^, ^36^, ^37^ Recent research has demonstrated that HLA-DR+ NK cells possess the capability to process and present specific antigens, leading to the activation and proliferation of T-cells.^38^ An in vitro experiment examining the induction of TT-specific CD4+ T-cell proliferation by NK cell clones revealed that HLA-DR+ NK cell populations presented TT with varying efficiencies, which depended on the intensity of HLA-DR expression and antigen concentration in the culture medium.^39^ Moreover, pre-activation of HLA-DR+ NK cells with IL-2 has been shown to significantly enhance CD3-induced CD4+ T-cell proliferation and IFN-γ production, thereby amplifying inflammatory and immune responses.^40^^,^^41^ Findings from this study indicate that an increased SSC-A level on HLA-DR+ NK cells is positively correlated with the risk of AR. A higher SSC-A level reflects greater internal complexity within HLA-DR+ NK cells, which may be linked to enhanced antigen processing and presentation capabilities. Although limited research has explored the role of the HLA-DR+ NK cell population in AR, it is speculated that these cells may contribute to the immune response by stimulating T-cell activation and proliferation releasing inflammatory cytokine.

Research into the relationship between these findings and AR is currently limited. Nonetheless, these findings suggest potential future research directions and treatments for AR. Key research directions include Elucidating the correlation between elevated CD3 expression and TCR signal intensity, and how this enhances the immunosuppressive function of specific CD8+ T-cell subsets to inhibit AR immune responses. Evaluating the efficacy of PD-1 agonists or PDL-1 upregulation therapies in suppressing Th2 cell activation and IL-4/IL-5/IL-13 secretion in AR models. Investigating the interaction between HLA-DR+ NK cells and Th2 cells that promotes the AR inflammatory cascade. Regarding treatment, potential strategies involve: Utilizing PD-1 agonists (e.g., low-dose antibodies) via local (nasal spray) or systemic administration to suppress the Th2 response and promote Tregs differentiation, thereby alleviating inflammation. Developing antibodies or small molecules to block HLA-DR-mediated antigen presentation and inhibit the immune response.

In this study, a large sample size was utilized to minimize the influence of confounding factors and provide a reliable estimation of causality between exposure factors and disease, mitigating the risk of reverse causality. However, certain limitations should be acknowledged. First, due to the limited number of genome-wide significant SNPs (p < 5 × 10^−8^), we applied a lenient threshold for selecting immune cell IVs (p < 5 × 10^−6^), potentially introducing weak instrument bias, which was mitigated through sensitivity analyses. Second, immune cell data and AR data were obtained from different studies, resulting in variations in quality control methods, sample sizes, and ethnic compositions, which may introduce potential biases. Third, despite multiple sensitivity analyses, pleiotropy, the effect of a single genetic variant on multiple phenotypes, may remain. Fourth, the GWAS data used in this study were derived from European populations, which may limit the generalizability of the findings to other ethnic groups.

## Conclusion

In summary, the comprehensive MR analysis provided evidence of a causal relationship between 38 immune cell phenotypes and AR in European populations. Specifically, SSC-A on HLA-DR+ NK cells was positively associated with the risk of AR, whereas CD3 on CD28+ CD45RA+ CD8+ T-cells and PDL-1 on IL14-IL16- were negatively with it. These findings offer a genetic perspective on the intricate relationship between immune cell phenotypes and AR, contributing to a deeper research direction involved in AR pathogenesis and highlighting potential directions for novel therapy strategies.

## ORCID ID

Yuhan Tan: 0009-0000-3618-9114

Xiaoyan Hu: 0000-0003-1241-5695

Jing Zhang: 0000-0002-4258-2278

Shuoyu Wan: 0009-0001-5270-7986

Liang Jiang: 0000-0001-6448-4479

## Funding

This study was supported by Sichuan Science and Technology Program (2025NSFSC2152) and Clinical medicine specialty of Southwest Medical University (2024LCYXZX44).

## Declaration of competing interest

The authors declare that the research was conducted in the absence of any commercial or financial relationships that could be construed as a potential conflict of interest.
